# One-step synthesis of amino-functionalized up-converting NaYF_4_:Yb,Er nanoparticles for *in vitro* cell imaging†

**DOI:** 10.1039/c8ra04178d

**Published:** 2018-08-01

**Authors:** Lidija Mancic, Aleksandra Djukic-Vukovic, Ivana Dinic, Marko G. Nikolic, Mihailo D. Rabasovic, Aleksandar J. Krmpot, Antonio M. L. M. Costa, Bojan A. Marinkovic, Ljiljana Mojovic, Olivera Milosevic

**Affiliations:** Institute of Technical Sciences of the Serbian Academy of Sciences and Arts Belgrade Serbia lidija.mancic@itn.sanu.ac.rs; Department of Biochemical Engineering and Biotechnology, Faculty of Technology and Metallurgy, University of Belgrade Serbia; Innovation Center of the Faculty of Chemistry, University of Belgrade Serbia; Photonic Center, Institute of Physics Belgrade, University of Belgrade Zemun Belgrade Serbia; Department of Chemical and Materials Engineering, Pontifical Catholic University of Rio de Janeiro Rio de Janeiro Brazil

## Abstract

The emerging up-conversion nanoparticles (UCNPs) offer a wide range of biotechnology applications, from biomarkers and deep tissue imaging, to single molecule tracking and drug delivery. Their successful conjugation to biocompatible agents is crucial for specific molecules recognition and usually requires multiple steps which may lead to low reproducibility. Here, we report a simple and rapid one-step procedure for *in situ* synthesis of biocompatible amino-functionalized NaYF_4_:Yb,Er UCNPs that could be used for NIR-driven fluorescence cell labeling. X-ray diffraction showed that UCNPs synthesized through chitosan-assisted solvothermal processing are monophasic and crystallize in a cubic α phase. Scanning and transmission electron microscopy revealed that the obtained crystals are spherical in shape with a mean diameter of 120 nm. Photoluminescence spectra indicated weaker green (^2^H_11/2_, ^4^S_3/2_ → ^4^I_15/2_) and stronger red emission (^4^F_9/2_ → ^4^I_15/2_), as a result of enhanced non-radiative ^4^I_11/2_ → ^4^I_13/2_ Er^3+^ relaxation. The presence of chitosan groups at the surface of UCNPs was confirmed by Fourier transform infrared spectroscopy, thermogravimetry and X-ray photoelectron spectroscopy. This provides their enhanced internalization in cells, at low concentration of 10 μg ml^−1^, without suppression of cell viability after 24 h of exposure. Furthermore, upon 980 nm laser irradiation, the amino-functionalized NaYF_4_:Yb,Er UCNPs were successfully used *in vitro* for labeling of two human cell types, normal gingival and oral squamous cell carcinoma.

## Abstract 2

The emerging up-conversion nanoparticles (UCNPs) offer a wide range of biotechnology applications, from biomarkers and deep tissue imaging, to single molecule tracking and drug delivery.

## Graphical abstract

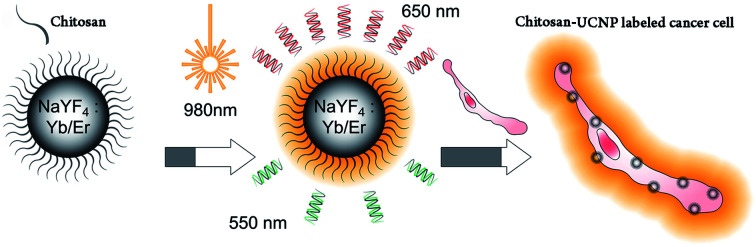

## Introduction

Nanotechnology research in the last few decades has been driven by both technological and fundamental interests, in an effort to develop advanced multifunctional biomaterials for a broad range of applications. It has been shown already that functionalized inorganic nanoparticles could be used as theranostic nanoplatforms when grafting of drugs/antigens is successfully completed at their surface.^1,2^ Particularly, tailored coupling of optically active lanthanide doped inorganic fluorides and oxides, that have the ability to convert long-wavelength near infrared (NIR) excitation into shorter-wavelength emission of visible light (up-conversion, UC), with a variety of biomolecules, generates hybrid nanoparticles which possess superior bioimaging and therapeutic characteristics.^3,4^ Compared with traditional fluorescent dyes, up-converting nanoparticles (UCNPs) offer several advantages, including excellent chemical and thermal stability, narrow-band emission, a large anti-Stokes shift and a long lifetime. The absence of photobleaching and blinking are other advantages that meet the requirements of background free detection in deeper tissues, as well as time-resolved imaging of morphological details from cells. Intrinsic optical properties of UCNPs originate from abundant energy states of lanthanide ions doped in a host matrix. The electron transitions between partially filled 4f orbitals which are effectively shielded by 5s and 5p are Laporte forbidden, so gaining of their intensity occurs through the mixing in higher electronic states of opposite parity, either by “vibronic coupling” or through the effect of a ligand field.^5^ To enhance probability of radiative transitions, at least two lanthanide ions (sensitizer and activator) are usually doped in host material with a strong crystal field and low phonon energy (like oxides and fluorides). The UC goes on through following mechanisms: excited state absorption (ESA), energy transfer (ET), photon avalanche (PA), cooperative energy transfer (CET) and energy migration-mediated up-conversion (EMU). For ET to occur, the excited energy levels of both ions must be resonant, and ions should be in close spatial proximity.^6^ For instance, ^2^F_5/2_ level of ytterbium resonates well with energy levels of erbium, thulium and holmium, so it is used as a very efficient sensitizer for achieving efficient UC in NaYF_4_ host. In Yb^3+^/Er^3+^ co-doped NaYF_4_ nanoparticles, green (520 nm and 540 nm) and red (660 nm) emissions are most commonly observed under 980 nm excitation, while violet emission at 415 nm is usually weakened. The emission is dependent on dopants concentration and crystal arrangement of the NaYF_4_ phase. In the structure of cubic α phase lanthanide and sodium ions occupy eight-coordinated cation site randomly, whilst in hexagonal β phase cation sites are of three types: a one-fold site occupied solely by lanthanides; a one-fold site occupied randomly by 1/2 lanthanides and 1/2 sodium; and a two-fold site occupied by sodium and vacancies stochastically.^7^ As a result, green and red emissions are both prominent in the spectra of cubic phase, whereas green dominates in spectra of hexagonal one.

The rapid progress in development of different protocols which give rise to the synthesis of monodisperse lanthanide doped NaYF_4_ UCNPs through decomposition of organometallic precursors, proposed initially by Mai *et al.*,^8^ is replaced nowadays with studies devoted to *in situ* obtaining of biocompatible UCNPs.^9,10^ This is due to the fact that synthesis from toxic organometallic, performed in an oxygen-free environment, must be followed by SiO_2_ encapsulation, ligands exchange/oxidation or by coating with a biocompatible polymer towards achieving a demanded chemical functionality for conjugation of the targeting molecular moiety. Although well established, the reproducibility of multiple steps involved in such synthesis is not trivial, since toxicity of UCNPs produced throughout is not easily predictable.^11^ In order to obtain physiologically stable NaYF_4_:Yb^3+^/Er^3+^ nanoparticles *in situ*, some biopolymers are already being used as surfactants during hydro/solvothermal synthesis. Wang *et al.*^12^ were the first who reported a simple one-step approach for the synthesis of hydrophilic UCNPs which comprises polyethylenimine (PEI), polyacrylic acid (PAA), polyvinylpyrrolidone (PVP) and polyethylene glycol (PEG) usage during hydrothermal treatment. Many years later it was shown that such functionalized particles could be easily conjugated to folic acid, a commonly used cancer targeting agent, and then loaded with doxorubicin hydrochloride to achieve pH-responsive release at target cells.^13^ Furthermore, PEGylated nanoparticles of α-NaYF_4_:Yb,Er, prepared under the cooperative influence of two ligands, demonstrated low cytotoxicity and excellent distribution in small animals.^14^ Under the guidance of the same concept we have shown that PEG, PVP and EDTA capped NaYF_4_:Yb,Er nano- and micro-particles could be easily obtained in a controlled manner through tuning of hydro/solvothermal processing conditions.^15,16^

In this study, amino-functionalized NaYF_4_:Yb,Er nanoparticles with a high degree of size uniformity and efficient up-conversion were prepared through solvothermal treating of rare earth nitrates in the presence of chitosan (CS). Chitosan is a linear polysaccharide composed of randomly distributed β-linked d-glucosamine and *N*-acetyl-d-glucosamine. Inter-dispersed acetamido groups, as well as an abundance of external –NH_2_ and –OH functional groups offer excellent biocompatibility and bio-reactivity, making it to be one of the most valuable polymer for medical and pharmaceutical applications. It is widely used today in encapsulation and controlled delivery of drugs, wound dressing, construction of contact lenses and artificial skin substitutes.^17,18^ An immense array of depicted benefits was recently complemented with its prominent antitumor activity.^19^ Lately, several reports related to chitosan coupling to inorganic UCNPs were also reported. Thus, an effective coating of DMSA-modified NaYF_4_:Yb/Er UC with folic acid–chitosan conjugates was achieved through a robust approach which comprised covalent bonding of amine groups with carboxyl groups located at particle surface.^20^ Besides, amphiphilic *N*-succinyl-*N*′-octyl chitosan modified UCNPs coupled with Zn(ii)-phthalocyanine photosensitizer, in form of novel drug delivery system, ZnPc-loaded SOC-UCNPs, demonstrated promising potential for NIR triggered photodynamic therapy of human breast adenocarcinoma.^21^ More recent studies shown that quaternized chitosan hydrogels incorporated with NaYF_4_:Er/Yb/Mn@photosensitizer-doped silica could be used for effective killing of both Gram-positive and Gram-negative bacteria,^22^ whilst spherical chitosan–NaYF_4_:Yb^3+^/Tm^3+^composite beads have excellent drug loading capacity and release performance upon near-infrared (NIR) laser irradiation.^23^ Although presented results provide evidence of the significant therapeutic effects, all of aforementioned hybrid UCNPs were actually obtained through multiple steps. It is worth noting that there is only one report, as far as the authors are aware of, on the usages of *O*-carboxymethyl chitosan during solvothermal preparation of NaYF_4_:Yb^3+^/Tm^3+^/Er^3+^ nanoparticles (UCNPs@OCMC), but successful staining of the HeLa cancer cells was achieved only after additional bio-conjugation of synthesized UCNPs@OCMC with folic acid.^24^

Hence, the main goal of the present study is *in situ* synthesis of chitosan functionalized NaYF_4_:Yb,Er nanoparticles capable of transforming continual NIR radiation into visible light and their successful utilization in visualization of the oral squamous cell carcinoma (OSCC). OSCC is the most common malignant tumor of the head and neck. Its incidence has increased in the recent years, thus development of a new contrast-enhanced agent useful for its detection at an early stage is essential. The potential cytotoxicity of the as-obtained UCNPs synthesized in this study was additionally tested against human gingival cells (HGC) isolated from healthy gingival tissue.

## Materials and methods

### Reagents and materials

Chitosan (low molecular weight, 50 000–190 000 Da), sodium fluoride (NaF, 99.99%), yttrium(iii) nitrate hexahydrate (Y(NO_3_)_3_*x*6H_2_O, 99.9%), ytterbium(iii) nitrate pentahydrate (Yb(NO_3_)_3_*x*5H_2_O, 99.9%), erbium(iii) nitrate pentahydrate (Er(NO_3_)_3_*x*5H_2_O,99.9%), anhydrous ethylene glycol (C_2_H_6_O_2_, 99.8%), phosphate-buffered saline (PBS), fetal bovine serum (FBS), dimethyl sulfoxide (DMSO), Dulbecco's Modified Eagle Medium (DMEM), penicillin–streptomycin (100 U ml^−1^), 3-(4,5-dimethylthiazol-2-yl)-2,5 diphenyltetrazolium bromide (MTT, 0.5 mg ml^−1^), paraformaldehyde (PFA) and Mowiol were all purchased from Sigma-Aldrich, St. Louis, USA. TrypLE Express enzyme, Gibco™ Dulbecco's modified Eagle's F12 medium (D-MEM/F12) and antibiotic/antimycotic solution (ABAM, 1%) were bought from Thermo Fisher Scientific. Deionized water was used throughout the experiments.

### Synthesis of amino modified NaYF_4_:Yb,Er

Monodispersed NaY_0.8_Yb_0.17_Er_0.03_F_4_ nanoparticles were synthesized using facile one-pot solvothermal synthesis. Stoichiometrically defined amounts of rare earth nitrates (5 mmol in total) were dissolved initially in 10 ml of water and then added to a chitosan solution (0.1 g CS in 15 ml of water). Obtained clear solution was then gradually dropped into NaF solution (1.75-fold excess, 10 ml) and mixed further with 35 ml of ethylene glycol (EG). Stirring of the mixture is performed until homogeneous transparent solution was obtained at pH = 4, then transferred into a 100 ml Teflon-lined stainless steel autoclave and sealed. Solvothermal treating was carried out at temperature of 200 °C (2 h) with a slow continual stirring (∼100 rpm). Afterwards, the autoclave was cooled to room temperature, the precipitate were centrifuged at 8000 rpm and then washed with ethanol three times. The as-obtained white powder was dried at 60 °C for 2 h.

### Characterization of amino-functionalized NaYF_4_:Yb,Er

Structural and morphological characteristics of amino-functionalized NaYF_4_:Yb,Er powder were obtained through the X-ray powder diffraction (XRPD), scanning and transmission electron microscopy (JEOL JSM-6701F SEM and JEOL JEM 2010 TEM), Fourier transform infrared spectroscopy (FTIR, Thermo Scientific Nicolet 6700 with a Smart iTR Diamond Attenuated Total Reflectance accessory) and thermogravimetric analysis (Perkin-Elmer Simultaneous Thermal Analyzer, STA 6000). The XRPD pattern was recorded using Bruker D8 Discovery equipped with a Cu-Kα source (*λ* = 1.5406 Å) with a step scan of 0.02° and accounting time of 5 s per step. Structure refinement was done in Topas 4.2 software^25^ using a fundamental parameter approach. The background was refined using a fifth-order Chebyshev polynomial. Refinement of the cubic phase was carried out in *Fm*-3*m* (no. 225) space group, starting from ICSD 60257 data. Isotropic size-strain analysis was performed using a predefined double-Voigt approach (volume weighted mean column height, FWHM based LVol). Due to the observed preferential orientation, the spherical harmonic formulation, also referred as ‘‘orientation distribution function”, is included in fitting of diffraction lines intensities. The size, shape and chemical purity of the nanoparticles were determined by SEM coupled with energy dispersive spectroscopy (EDS). The SemAfore 5.21 JEOL software was used to construct histogram of particle size from backscatter SEM images presenting more than 300 particles. Dynamic light scattering measurements of hydrodynamic radius (*R*_H_) were performed on a Malvern Zetasizer Nano ZS in the de-ionized water and medium used for testing of the cell viability and imaging. For that purpose UCNPs were dispersed at the concentration of 1 mg ml^−1^ and passed through a 0.45 μm cellulose syringe filter before DLS measurements. For TEM analysis nanoparticles were sonicated 20 min in isopropyl alcohol and dropped directly on lacey carbon film supported on a Cu grid. Confirmation of the crystal structure was carried out using selected area electron diffraction (SAED) and Fourier processing in Digital Micrograph 3.7.4 (Gatan Inc.) software. Presence of the chitosan ligands on the nanoparticles surface was investigated by FTIR, TG and XPS analyses. FTIR spectrum was recorded using typically 128 scans at the resolution of 4 cm^−1^. TGA was conducted in nitrogen flux (100 ml min^−1^) in the temperature range between 30 and 730 °C, applying a heating rate of 5 °C min^−1^. XPS was carried out using an Alpha 100 hemispherical analyser from VG thermo and the *K*_α_ line from Mg (1486.6 eV) radiation. Photoluminescence spectrum was recorded at room temperature using Spex Fluorolog with C31034 cooled photomultiplier under diode laser excitation at 980 nm, and based on it CIE chromaticity coordinates were calculated.

### OSCC and HGC cultures

Healthy gingival and tumor tissues were obtained from the patients at the Clinic of Maxillofacial Surgery of School of Dental Medicine, University of Belgrade immediately after surgical procedure. Signed informed consent approval from each patient was assured prior to participation in this study. Experiments were authorized by the Ethical Committee of the School of Dental Medicine, University of Belgrade (resolution 36/31).

Tumor cell lines were derived from tumor tissue taken from localized squamous cell carcinoma of the oral tongue. Preparation of the cell culture was performed using slightly modified procedure of Pozzi *et al.*^26^ Briefly, DMEM supplemented with 20% fetal bovine serum (FBS) and 100 U ml^−1^ penicillin–streptomycin was used for tissue transport. The cells isolated from minced tissue were seeded onto T25 cell culture flasks and grown in DMEM supplemented with 10% FBS and 100 U ml^−1^ penicillin–streptomycin. Incubation was performed at 37 °C in a humidified atmosphere of 5% CO_2_. The medium was changed thrice weekly and cells were passaged prior to reaching 80% confluence. To avoid fibroblast contamination, brief exposure to TrypLE Express (Thermo Fisher Scientific, Waltham, USA) was performed. OSCC used in this study were obtained after the third passage.

Human gingival tissues were obtained from three different, healthy patients, aged 19–25 years, during extraction of the impacted third molar. The gingival tissue was transported in Gibco™ D-MEM/F12 supplemented with 20% FBS and 1% ABAM solution. The gingival tissue was rinsed in PBS and subjected to outgrowth isolation method. Tissue was minced into approximately 1 mm^2^ fragments, and placed in 25 cm^2^ culture flasks with DMEM/F12 supplemented with 10% FBS and 1% ABAM. Incubation was performed at 37 °C in a humidified atmosphere of 5% CO_2_. The cells were allowed to reach 80% confluence prior to passage. The medium was changed every 2–3 days. HGC used in this study were obtained after the second passage.

### Cytotoxicity assay

MTT assays were carried out to evaluate the potential cytotoxicity of amino-functionalized NaYF_4_:Yb,Er in both, HGC and OSCC. Cells were seeded into a 96-well cell culture plate at 10^4^ per well and incubated at 37 °C in humidified 5% CO_2_ atmosphere. Then, aseptically weighted UCNPs in concentrations of 10, 25 and 50 μg ml^−1^ were dispersed in sterile water by sonication (3 min). After 24 h hours of cells incubation 100 μl of UCNPs were added in each plate. Incubation of UCNPs with the cell cultures was stopped after 24 h by discarding of spent media, and medium containing MTT (0.5 mg ml^−1^) was added to each well. After additional incubation for 4 h, supernatant was discarded and the precipitated formazan crystals were dissolved by DMSO (100 μl) under shaking at 37 °C for 20 min. Optical density was measured at 540 nm using ELISA microplate reader (enzyme-linked immunosorbent assay) RT-2100c, Rayto, China. Three wells without UCNPs were used as a control group. All experiments were performed in triplicate and repeated three times in the independent experiments. Cell viability, expressed by the ratio of absorbance of the cells incubated with UCNPs to that of the cells incubated with culture medium only, was given in diagram as the mean ± standard deviation (SD).

### Cell imaging by laser scanning microscopy

For the visualization of UCNPs uptake by cells 10 μg ml^−1^ of sterile UCNPs suspension was filtered through 0.45 μm syringe filter to separate agglomerates that could provoke saturation during imaging. Sterilized 22 × 22 mm glass coverslips were placed in 6-well plates and 10^4^ of cells were seeded per coverslip. Incubation was performed at 37 °C in humidified 5% CO_2_ atmosphere. The next day cells were exposed to UCNPs and incubated for another 24 h. Coverslips with adherent cells were gently rinsed with fresh PBS twice and fixed with 4% PFA for 20 min. PFA residue was then washed by PBS (3 × 3 min), coverslips were dried, 10 μl of Mowiol was placed on fixed cells, and coverslips were placed on microscopic slides with cells positioned in between. Samples were stored in a dark until they were observed under laser scanning microscopy.

The homemade nonlinear laser scanning microscope used in this study was described in detail elsewhere.^27^ The Ti:Sapphire laser (Coherent, Mira 900-F) was used as a laser light source operating either in femto-second (FS) pulse mode or continuous wave (CW) mode. FS mode at 730 nm was used for unlabeled cell imaging since it enables two photon excitation of cells auto-fluorescence. Please note that two-photon excitation is considered here as excitation of the molecule with no intermediate levels between ground and excited state and it is not related to UC process. From the other hand, 730 nm light do not interact with UCNPs. The CW radiation at 980 nm was used for the excitation of UCNPs in cells. Analogously to the previous case, CW 980 nm light cannot excite any other molecule except UCNPs. Due to the long UCNPs lifetime, the acquisition time at a single point has to be reduced during scanning in order to extend pixel dwell time. Hence, the pixel dwell time was several times longer than fluorescence lifetime. The laser focusing and collection of the fluorescence during cell imaging were done using 40 × 1.3 oil immersion objective (Carl Zeiss, EC Plan-NEOFLUAR). A visible interference filter (415–685 nm) positioned in front of detector was used to remove scattered laser light. Thus, the whole visible range has been detected either for auto-fluorescence from cells or for up-conversion from amino-functionalized NaYF_4_:Yb,Er UCNPs in cells.

## Results and discussion

**Fig. 1:**
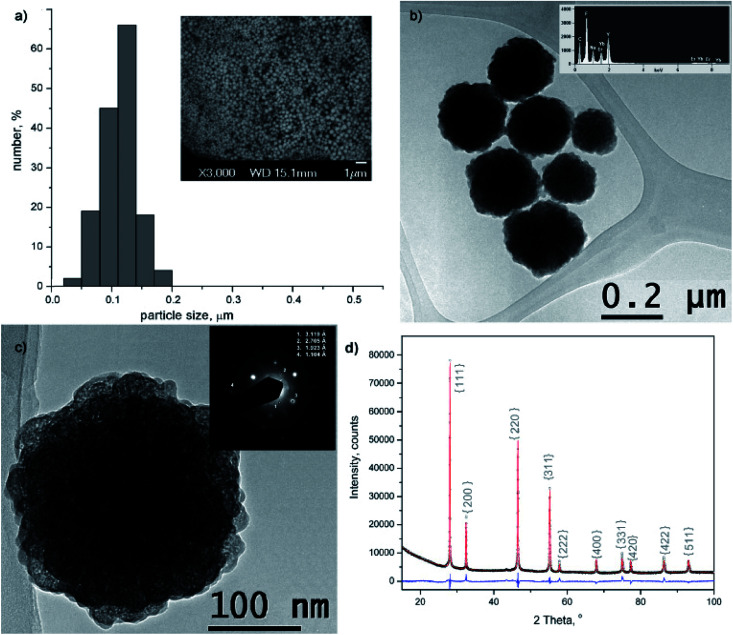
Particle size distribution histogram (a) and TEM images (b and c) of amino-functionalized NaYF_4_:Yb,Er UCNPs. Corresponding SEM, EDS, SAED and FFT were given as insets in (a), (b) and (c), respectively; XRPD pattern of amino-functionalized NaYF_4_:Yb,Er (black), refined structure (red) and difference curve (blue) is presented in (d). Miller indices are indicated in {} by gray marks.

The morphology and size of amino-functionalized NaYF_4_:Yb,Er UCNPs was evaluated by scanning electron microscopy (Fig. 1a). The SEM image showed that the as-obtained nanoparticles were spherical in shape, monodispersed and without obvious aggregation. Particle size varied between 50 and 200 nm and the bulk of the particles (>65%) are with diameter of 120 nm. The purity and expected chemical composition of the as-obtained NaYF_4_:Yb,Er phase were confirmed by an energy dispersive spectrometer coupled to TEM, Fig. 1b. Based on the elemental analysis (inset at Fig. 1b), it is evident the presence of all constituting elements: sodium (Kα at 1.041), yttrium (Lα at 1.92 keV), ytterbium (Lα at 7.414 and Mα at 1.521 keV), erbium (Lα at 6.947 and Mα at 1.404 keV) and fluorine (Kα at 0.677). TEM/SAED analyses, Fig. 1c, showed a mixture of distinct spots and rings in the SAED pattern which correspond to *d* values of 3.119, 2.705, 1.923 and 1.104 Å and match fine with (111), (200), (220) and (423) crystal planes of the cubic α NaYF_4_ phase (JCPDS 77-2042). Coexistence of much smaller crystallites of α phase at the UCNPs surface is evident from Fig. 1c and HRTEM/FFT images, Fig. S1.†

The XRPD pattern of the as-obtained sample was indexed to cubic α structure of NaYF_4_:Yb,Er phase (JCPDS no. 77-2042, *a* = 5.47 Å, *V* = 163.67 Å), Fig. 1d. However, structural refinement confirmed the coexistence of two different particle populations (two NaYF_4_:Yb,Er phases were used to adjust experimental pattern), both adopting the same *Fm*-3*m* space group with a similar unit cell parameters (Å): *a*_1_ = 5.51830(9) and *a*_2_ = 5.53074(1), and a quite different crystallite size (nm): 84(4) and 12(1), respectively. Calculated crystallites sizes were in agreement with the size observed during TEM analysis. Small change of the refined unit cell volume (168.04 Å) might be due to the Yb^3+^ and Er^3+^ incorporation at Y^3+^ site. Occupation of 0.41(1) for Y^3+^ site, correlates well with the nominal NaY_0.8_Yb_0.17_Er_0.3_F_4_ composition (the value of 0.5 corresponds to the full occupation of the general site occupied by rare earths in the cubic NaYF_4_ phase), whilst *R*_Bragg_ value of 5.7 confirmed good agreement between observed and computed patterns. The relevant information about Y^3+^ sites occupancy with rare earth ions in the phase with the smaller crystallite size were not acquired, due to the fact that better refinement was achieved when fixed values of occupation factor were used.

**Fig. 2:**
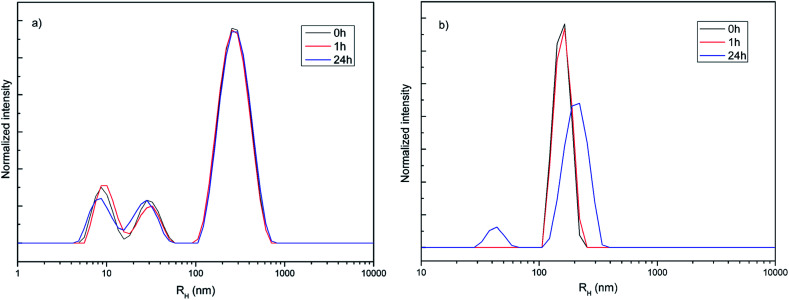
Hydrodynamic radius distribution over time of amino-functionalized NaYF_4_:Yb,Er UCNPs (1 mg ml^−1^) in medium used for testing of cell viability and imaging (a) and deionized water (b).

Hydrodynamic radius (*R*_H_), polydispersity index (PDI) and stability of amino-functionalized NaYF_4_:Yb,Er UCNPs colloids over time were estimated from dynamic light scattering measurements, Fig. 2. As one could see, the coexistence of different particle populations (one sized up to 30 nm and another bigger than 100 nm), indicated by TEM an XRPD analysis is well preserved in medium solution over time confirming the long-term stability of this colloid (Fig. 2a). Since DLS measures actual particle size plus thickness of the strongly bounded solvent shell around particles, obtained average values are different (60 nm with PDI 1 in medium, and 311 nm with PDI 0.34 de-ionized water, respectively) than determined ones from TEM images (120 nm). While average *R*_H_ and PDI of amino-functionalized NaYF_4_:Yb,Er UCNPs in medium stay unchanged with time, slight increase of both parameters were detected after one hour (380 nm and PDI 0.4) in water (Fig. 2b). Decrease of *R*_H_ to 211 nm (PDI 0.34) after 24 h is observed and is due to appearance of a significant fraction of clusters (∼50 nm) composed from nanoparticles sized up to 20 nm.

**Fig. 3:**
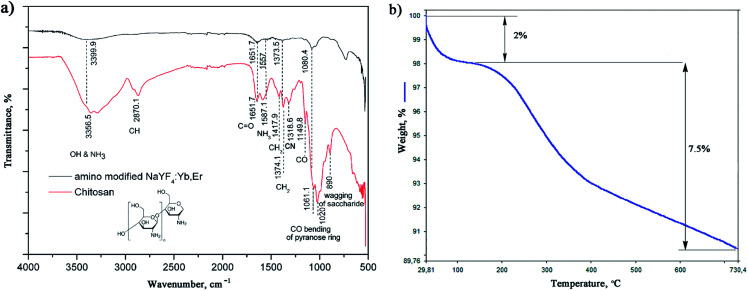
FTIR of chitosan and amino-functionalized NaYF_4_:Yb,Er (a) TGA of amino-functionalized NaYF_4_:Yb,Er UCNPs (b).

The successful *in situ* modification of the UCNPs surface with chitosan ligands was confirmed by FTIR spectroscopy (Fig. 3a). In accordance to the literature^28–30^ observed bands in spectrum of pure chitosan were classified as follows: broad band in the range from 3500 to 3000 cm^−1^ is due to stretching vibration of OH groups, which partially overlaps stretching vibration of amine N–H; band at 2870.1 cm^−1^ is due stretching of C–H bond in –CH_3_; band at 1651.7 cm^−1^ corresponds to vibrations of carbonyl bonds of the amide group CONHR (C=O stretching, secondary amide); band at 1587.1 cm^−1^ is due to protonated amine stretching; bands at 1417.9 cm^−1^ and 1374.1 cm^−1^ are due CH_3_ and CH_2_ bending vibrations; band at 1318.6 cm^−1^ is associated to the amide III (C–N stretching) and CH_2_ wagging; band at 1149.8 cm^−1^ reflects asymmetric vibration of C–O group, whilst band at 1060.156 cm^−1^ is assigned to CO bending vibration of pyranose ring. The small band at 890 cm^−1^ corresponds to wagging of the saccharide structure of chitosan. The FTIR spectrum of the as-synthesized UCNPs showed a decrease in adsorption and slight shifting of chitosan related bands at: 3399.9 cm^−1^ (O–H and amine N^3+^–H group stretching), 1651.7 cm^−1^ (C=O stretching), 1557 cm^−1^ (protonated amine stretching), 1373.5 cm^−1^ (CH_2_ bending) and 1080.4 cm^−1^ (CO bending) implying their existence on the UCNPs surface. This confirmed that chitosan-assisted solvothermal synthesis of NaYF_4_:Yb,Er was an effective way for *in situ* obtaining of biocompatible UCNPs.

The TGA also show chitosan presence at the UCNPs surface, Fig. 3b. TGA curve shows total weight loss of 9.5% in the temperature region from 30–730 °C. The initial weight loss of 2% (<200 °C) is ascribed to the dehydration of adsorbed moisture and possible ethanol impurity, whilst the more intense loss of 7.5% at higher temperatures is, predominantly, due to the vaporization of the chitosan groups present on the UCNPs surface. In accordance to literature, the thermal degradation of chitosan in nitrogen is an one-step reaction which starts at 326.8 °C.^31^ Meanwhile, a total mass loss of *ca.* 2–4% has been reported for bare NaYF_4_:Yb,R (R: Pr, Nd, Sm, Eu, Tb, Dy or Er) UCNPs owning to the removal of water, ethanol and slow evaporation of NaF in the same temperature range.^32,33^

XPS analysis was also used to verify surface chemical composition of the amino-functionalized NaYF_4_:Yb,Er UCNPs. All of the lanthanide elements, as well as, Na, F, C, N and O are detected in XPS spectrum, Fig.S2.† The peak at 1073.5 eV is assigned to the binding energy of Na 1s; peaks at 160.96, 174.29 and 185.72 eV were assigned to the binding energies of Y 3d, Er 4d and Yb 4d respectively; and peak at 685.71 eV is related to F 1s.^34,35^ Peaks of C 1s, O 1s and N 1s were further decomposed in the fine-scan mode to confirm the bonding of chitosan at the NaYF_4_:Yb,Er UCNPs surface. The C 1s peak showed three components: at 284.8 eV, typical of carbon bonding to carbon and hydrogen [C–(C,N)]; at 282.92 eV due carbon bonding to oxygen or nitrogen [C–(O,N)]; and at 286.59 eV typical for acetal or amide group [O–C–O, N–C=O]. The O 1s contributions at 532.70 and 531.4 eV were due to oxygen of the polysaccharide backbone and amide respectively.^36^ The first two N 1s contributions at 399.71 and 397.9 eV confirm coexistence of the non-protonated and protonated amine groups, implying that approximately half of chitosan terminal amine groups are covalently bounded at UCNPs surface.

**Fig. 4:**
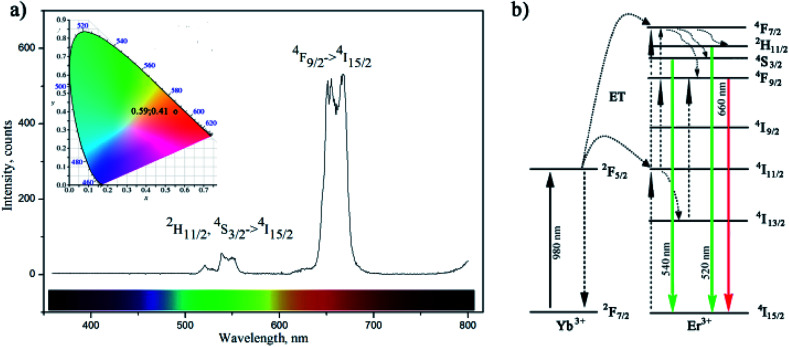
Up-conversion luminescence of amino-functionalized NaYF_4_:Yb,Er UCNPs upon excitation at 980 nm with corresponding CIE diagram given as inset (a) and energy level diagram of the Er^3+^–Yb^3+^ couple (b).

The up-conversion luminescence spectra of the amino-functionalized NaYF_4_:Yb,Er UCNPs is given at Fig. 4a. It can be split into two emission segments, a green region of 520–550 nm and a red region of 630–690 nm, attributed to ^4^S_3/2_ → ^4^I_15/2_ and ^4^F_9/2_ → ^4^I_15/2_ transitions of Er^3+^ ions, respectively. According to energy transfer and relaxation pathways depicted at the energy level diagram of the Er^3+^–Yb^3+^ couple (Fig. 4b), both were determined by the non-radiative decay from ^4^F_7/2_ excited state.

Upon the 980 nm excitation, Yb^3+^ absorbs energy and promotes ^2^F_7/2_ → ^2^F_5/2_ transitions. Afterward, it resonantly transfers the energy to the ^4^I_11/2_ state of the neighbouring Er^3+^ ion. The ^4^I_11/2_ level of Er^3+^ ion can be also populated by direct excitation of Er^3+^ ion from its ^4^I_15/2_ ground state. Thus, the higher excited states of Er^3+^, ^4^F_7/2_ and ^4^F_9/2_, will be further populated either through energy transfer from another excited Er^3+^ ion which is in close proximity, or through a two-step energy transfer from Yb^3+^ to the neighbouring Er^3+^ ions. The populated ^4^F_7/2_ level of Er^3+^ then relaxes non-radiatively to the ^2^H_11/2_ and ^4^S_3/2_ levels, and further radiatively to the ground ^4^I_15/2_ state generating green emissions at 520 nm (^2^H_11/2_ → ^4^I_15/2_) and 540 nm (^4^S_3/2_ → ^4^I_15/2_). Meanwhile, red emission appears due to the ^4^F_9/2_ → ^4^I_15/2_ de-excitation, which could be additionally intensified by the non-radiative ^4^F_7/2_ → ^4^F_9/2_ relaxation. Therefore, green and red emissions were obtained simultaneously through two-photon UC processes. Furthermore, due to the enhanced non-radiative relaxation of ^4^I_11/2_ → ^4^I_13/2_ in nanocrystals^16,31^ which proceeds to the direct population of ^4^F_9/2_ level emission of red/orange light (defined by CIE 0.59, 0.41) was observed by bare eyes at room temperature. Photostability of amino-functionalized up-converting NaYF_4_:Yb,Er nanoparticles emission was also traced during 1 h, Fig. S3.† As one could see at Fig. S3,† exceptionally stable UC luminescence signal was recorded.

**Fig. 5:**
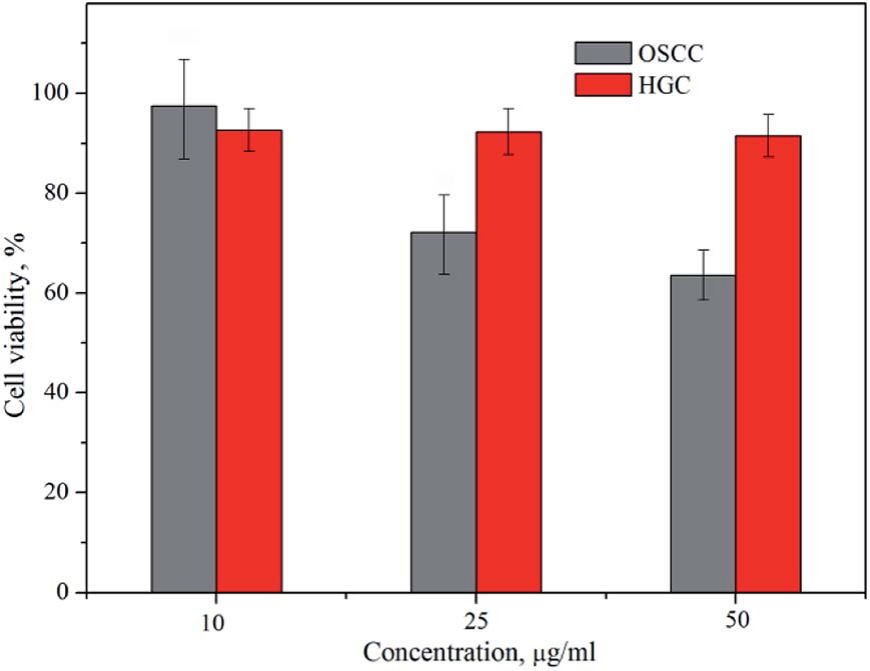
Cytotoxicity assays of the amino-functionalized NaYF_4_:Yb,Er UCNP in OSCC and HGC after 24 h exposure.

The viability of OSCC and HGC following 24 h incubation with the amino-functionalized NaYF_4_:Yb,Er UCNPs, expressed in terms of percentages evaluated through comparison to the number of surviving cells in the control group, was determined by MTT assay (Fig. 5). One could see that viability of HGC was highly preserved after 24 h exposure, being above 90% for all examined concentrations of UCNPs. However, viability of OSCC was found variable with the increase of UCNPs concentrations. Only in the case of the lowest concentration non-significant cytotoxicity (*i.e.* viability of 98%) was detected, whilst at higher UCNPs concentrations noteworthy cytotoxicity was observed, as reflected in a concentration dependent decrease in the percentage of viable cells up to the value of 66% for 50 μg ml^−1^. Previous study reported low cytotoxicity of the ZnPc-loaded SOC-UCNPs toward the adenocarcinoma cells for concentrations up to 200 μg ml^−1^.^21^ Also, insignificant difference in HeLa carcinoma cell viability (∼85%) has been observed when the concentrations of UCNPs@OCMC went up to 200 μg ml^−1^.^24^ When compared with latter two, the results obtained in this study are unanticipated and indicate certain theranostic effect of amino-functionalized NaYF_4_:Yb,Er UCNPs toward OSCC at much lower doses.

**Fig. 6:**
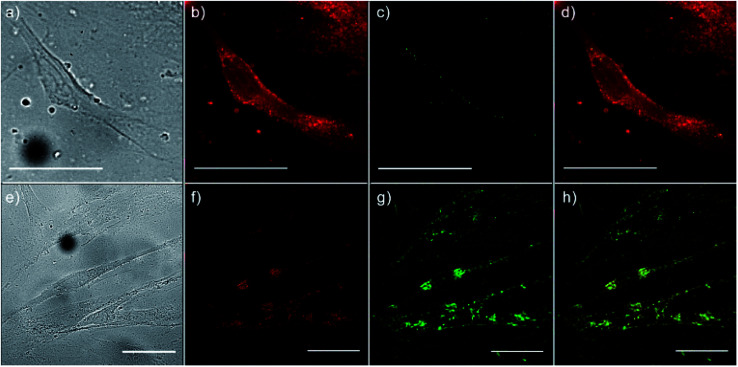
Laser scanning microscopy images of OSCC (top row) and HGC (bottom row) following 24 h incubation with 10 μg ml^−1^ of amino-functionalized NaYF_4_:Yb,Er UCNPs; bright field image of cells (a and e), cells auto-fluorescence upon femto-second excitation at 730 nm (b and f), image of the amino-functionalized NaYF_4_:Yb,Er UCNPs upon CW excitation at 980 nm (c and g), and their positioning in cells, revealed through co-localization of the cell auto-fluorescence and the UCNPs emission (d and h); the scale bars correspond to 50 μm.

To monitor the intracellular uptake and non-specific cell labelling *in vitro*, 10 μg ml^−1^ of amino-functionalized NaYF_4_:Yb,Er UCNPs were incubated with OSCC and HGC and after 24 h laser scanning microscopy was performed. Images of the OSCC are shown at Fig. 6, top row. Fig. 6a shows bright field image of the cell, a pseudo color image of the cell auto-fluorescence upon femto-second excitation at 730 nm is shown at Fig. 6b, whilst the pseudo color image of the amino-functionalized NaYF_4_:Yb,Er UCNPs upon CW excitation at 980 nm is given in Fig. 6c.

Overlapping the last two images (Fig. 6d), revealed that the UCNPs (green fluorescence spots) are positioned inside the cell, mainly in the cytoplasmic area adjacent to the plasma membrane. Images of the HGCs are shown in bottom row of Fig. 6 following the same scanning procedure. As in a previous case, successful internalization of UCNPs in the cytoplasmic region of cells was achieved without disturbing cell nuclei. Since no auto-fluorescence was observed from cells upon NIR excitation (Figs. 6c and g), successful cells visualization with UCNPs demonstrated the possibility of utilizing the amino-functionalized NaYF_4_:Yb,Er nanoparticles for nonspecific cell labelling. Furthermore, abundance of the chitosan ligands present at their surface (particularly amino groups), make them accessible for further conjugation with anti-cancer drugs, monoclonal antibodies or photosensitizes towards developing of specific theranostic agents.^37^ As it is pointed out before, in prior studies which involved UCNPs surface modification with chitosan, multistep procedures were used to achieve the benefits of both, biocompatibility and near-infrared triggered up-conversion in cells. We believe that facile approach presented in this study may be extended to the synthesis of UCNPs with other biocompatible ligands too, raising at that way their potential use in biomedicine.

## Conclusions

Monodisperse, hydrophilic and biocompatible NaYF_4_:Yb,Er UCNPs were synthesized *in situ* using chitosan-assisted solvothermal synthesis. Spherical particles sized around 120 nm contain a single crystal structure of a cubic α phase (*Fm*-3*m*) and emit intense orange light (CIE 0.59, 0.41) upon 980 nm laser excitation. Due to the presence of amino functional groups at their surface, NaYF_4_:Yb,Er UCNPs presented suitable properties for application in non-specific *in vitro* cell labelling. The superior biocompatibility detected toward normal human gingival cells with regard to oral squamous cell carcinoma, under similar cellular internalization, indicates their great potential in diagnostic and cancer therapy, particularly in a deeper tissue up to the penetration depth of NIR light.

## Conflicts of interest

There are no conflicts of interest to declare.

## Supplementary Material

RA-008-C8RA04178D-s001
