# Supplementary material for: One-step synthesis of amino-functionalized up-converting NaYF4:Yb,Er nanoparticles for in vitro cell imaging
Source: RSC Adv. 2018 Aug 1;8(48):27429–37. doi: 10.1039/c8ra04178d (PMC9083799; doi:10.1039/c8ra04178d)
Supplement: RA-008-C8RA04178D-s001 [file RA-008-C8RA04178D-s001.pdf]

Electronic Supplementary Material (ESI) for RSC Advances.
This journal is © The Royal Society of Chemistry 2018

Supplementary file

One-step synthesis of amino-functionalized up-converting $NaYF_4$:Yb,Er nanoparticles for *in vitro* cell imaging

Lidija Mancic, Aleksandra Djukic-Vukovic, Ivana Dinic, Marko G. Nikolic, Mihailo D. Rabasovic, Aleksandar J. Krmpot,
Antonio M.L.M. Costa, Bojan A. Marinkovic, Ljiljana Mojovic, Olivera Milosevic

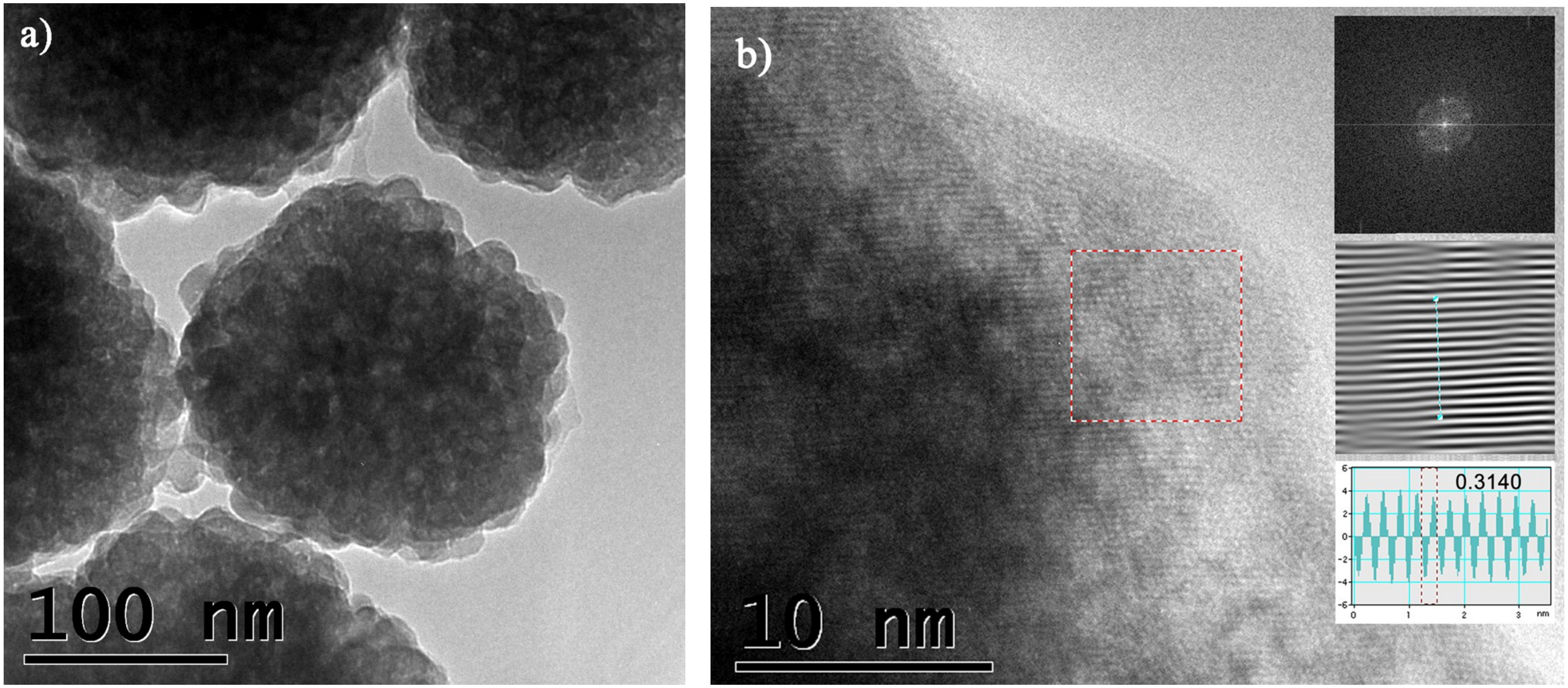

Fig. S1. TEM (a) and HRTEM (b) images of amino modified $NaYF_4$:Yb,Er UCNPs. Corresponding FFT/IFFT given as insets in b, confirms that much smaller crystallites notable at the particles surfaces (a) revealed periodic array of cubic α phase, (111) plane with *d* value of 3.140 Å

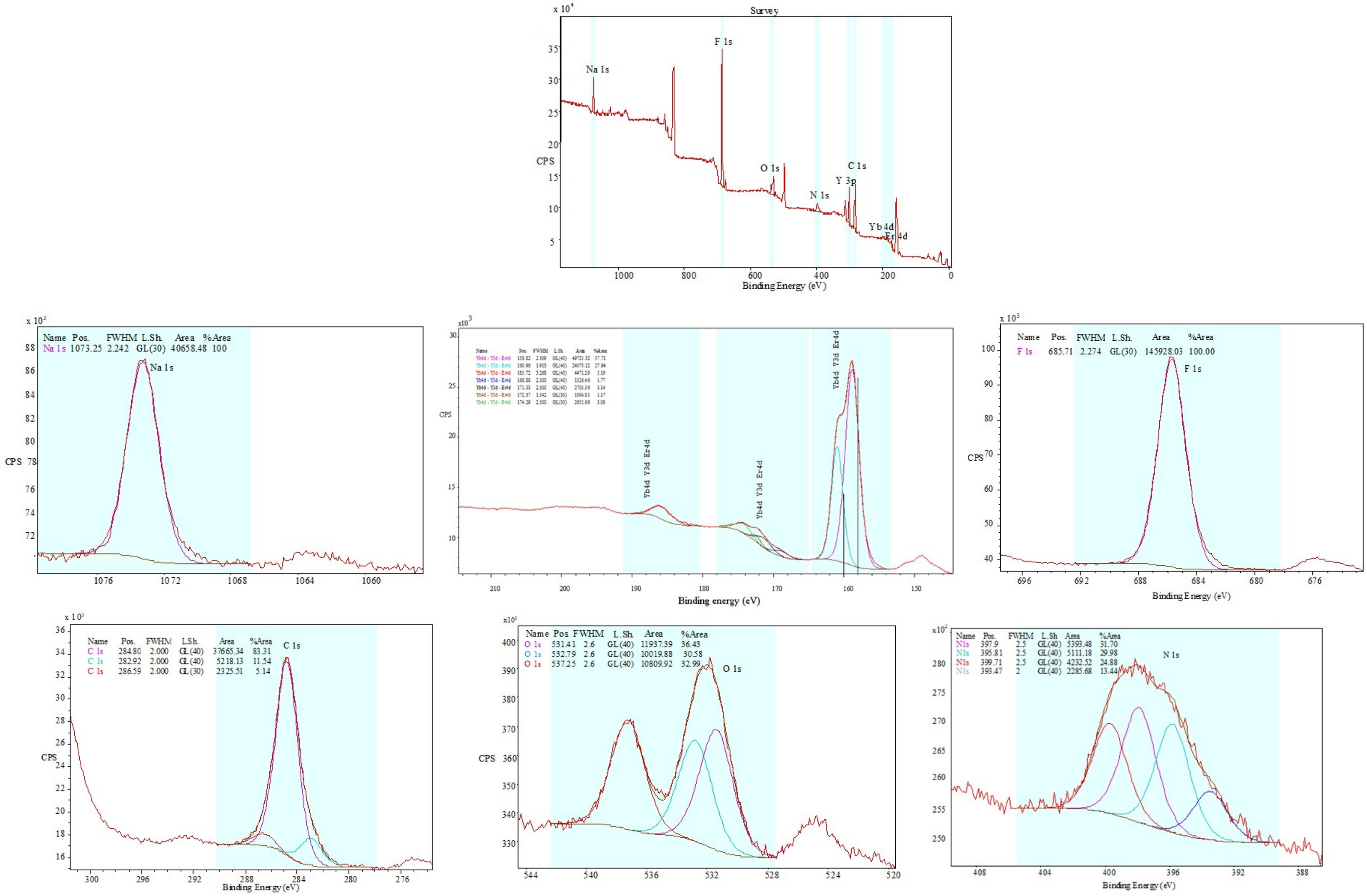

Fig. S2. XPS spectrum of amino-functionalized $NaYF_4$:Yb,Er UCNPs: survey spectrum and fine-scan spectra of Na 1s, Y 3d, Yb 4d, Er 4d, F 1s and decomposed ones of C 1s, O 1s and N 1s.

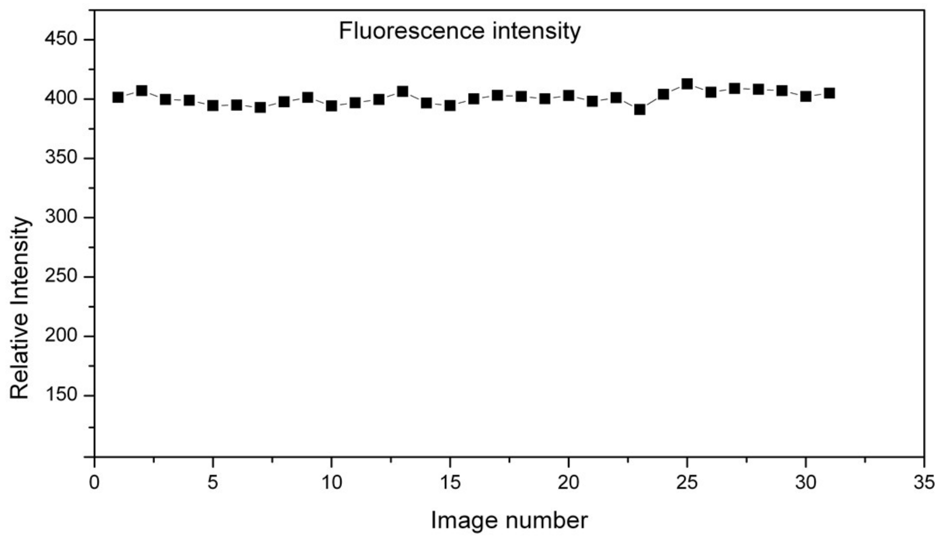

Fig. S3. Photostability of amino-functionalized up-converting $NaYF_4$:Yb,Er nanoparticles. The emission intensity was traced during 1h
